# Ciprofol provides safer sedation than propofol in minor gynecologic surgery: evidence from a randomized controlled trial

**DOI:** 10.1590/1806-9282.20251801

**Published:** 2026-06-15

**Authors:** Wenbo Huang, Qingtong Chen, Dashuo Peng, Yanhui Li, Wenyang Liu, Sijin Peng, Guiling Xie

**Affiliations:** 1 Heyuan People's Hospital, Department of Anesthesiology – Heyuan, China.; 2 Heyuan People's Hospital, Department of Gynaecology – Heyuan, China.

**Keywords:** Ciprofol, Propofol, Gynecologic surgery, Sedation

## Abstract

**OBJECTIVE:**

The quality of sedation critically affects the success and safety of minor gynecologic surgeries. Ciprofol, a novel analog of propofol, offers rapid onset and recovery with a potentially improved safety profile. This study compared the efficacy and safety of ciprofol and propofol for sedation during minor gynecologic surgery.

**METHODS:**

In this single-center, randomized, double-blind trial, 342 patients scheduled for minor gynecologic procedures were assigned to receive either ciprofol or propofol for anesthesia induction and maintenance. The primary outcomes were anesthesia onset time and surgical success rate. Secondary outcomes included recovery time, hemodynamic stability, body movement, injection pain, satisfaction ratings, and adverse events such as respiratory depression and hypotension.

**RESULTS:**

Both agents achieved a 100% anesthesia success rate, with comparable onset times (ciprofol: 37.2±10.4 s vs. propofol: 38.7±11.2 s; p=0.214). Ciprofol required a fivefold lower total dose (68.5±31.7 mg vs. 359.6±164.6 mg; p<0.001) and demonstrated superior safety, including fewer body movements (2.4 vs. 15.3%), absence of injection pain (0 vs. 36.5%), and reduced hypotension (10.1 vs. 41.2%; all p<0.001). Satisfaction was higher among surgeons and patients receiving ciprofol.

**CONCLUSION:**

Ciprofol provides anesthesia efficacy equivalent to propofol with significantly enhanced safety and tolerance, supporting its use as a preferred agent for outpatient gynecologic surgery.

## INTRODUCTION

In recent years, the pursuit of comfort-oriented medical care has led to the growing adoption of painless diagnostic and therapeutic procedures. Minor gynecologic surgery, characterized by minimal trauma and short operative duration, has become increasingly common. However, the severe pain associated with these procedures often provokes anxiety and fear, which may complicate surgery and necessitate effective anesthetic intervention. Common anesthetic methods for minor gynecologic surgery include local anesthesia, regional block, spinal anesthesia, and general anesthesia. Among these, propofol combined with opioids remains the most widely used regimen owing to its pharmacological advantages, such as rapid onset, predictable metabolism, and strong controllability^1^. Despite these benefits, propofol is associated with notable adverse effects, including circulatory suppression, respiratory depression, injection pain, and propofol infusion syndrome. Therefore, the search for alternative anesthetics with improved safety profiles has become a priority.

Ciprofol, a novel 2,6-disubstituted phenol derivative, is a structural analog of propofol that exhibits stronger binding affinity to the γ-aminobutyric acid A (GABA_A_) receptor. It has been developed for procedural sedation, induction and maintenance of general anesthesia, and intensive care unit (ICU) sedation. Previous studies have demonstrated that ciprofol at doses of 0.4–0.6 mg/kg produces comparable sedation and recovery profiles to propofol at 1.5–2.5 mg/kg, indicating a wider safety margin^2^. Other investigations have shown that ciprofol possesses a potency ratio of approximately 1/4 to 1/5 compared with propofol and is associated with a lower incidence of respiratory-related adverse events (AEs) and injection pain^3^. Similarly, Luo et al. reported that ciprofol exhibited equivalent efficacy to propofol at one-fifth the dose, with superior safety reflected in fewer drug-related adverse reactions and reduced lipid intake during fiberoptic bronchoscopy and ICU sedation^4^. Clinical trials have further confirmed that ciprofol is well tolerated during the induction and maintenance of general anesthesia, resulting in decreased hemodynamic fluctuations, fewer AEs, and significantly alleviated injection pain^5^.

Although recent studies have evaluated ciprofol in endoscopy, bronchoscopy, and general anesthesia, clinical evidence specifically supporting its use in minor gynecologic surgery remains limited. This surgical setting requires rapid anesthetic onset, stable hemodynamics, minimal respiratory depression, and a high level of patient comfort—areas in which propofol, despite its widespread use, is often constrained by adverse effects such as hypotension, respiratory suppression, and injection pain. Given ciprofol's higher GABA_A_ receptor affinity, wider safety margin, and markedly lower incidence of vascular irritation, it may offer significant advantages over propofol. However, direct comparative data in outpatient gynecologic procedures are scarce.

Therefore, we designed a single-center, randomized, double-blind, active-controlled, non-inferiority trial to compare the efficacy and safety of ciprofol versus propofol in patients undergoing minor gynecologic surgery, with the aim of providing high-quality evidence to guide safer and more effective anesthetic selection in outpatient gynecologic practice.

## METHODS

### Patient selection and eligibility criteria

This study enrolled female patients scheduled for minor gynecologic surgery under sedation. Eligible participants were between 18 and 65 years of age and classified as American Society of Anesthesiologists (ASA) physical status I or II. Physiological stability was ensured by the following inclusion criteria: body mass index (BMI) 18–30 kg/m^2^, respiratory rate (RR) 10–24 breaths/min, pulse oxygen saturation (SpO_2_) ≥95%, systolic blood pressure (SBP) 85–140 mmHg, diastolic blood pressure (DBP) 50–90 mmHg, and HR 50–100 beats/min.

Patients were excluded if they had a history of alcohol abuse, a known allergy to general anesthetics, or long-term use of sedative-hypnotic or anti-anxiety medications. Those with unregulated or malignant hypertension, severe ischemic heart disease, or significant hepatic or renal dysfunction were also excluded. Pregnant or lactating women, patients with recent respiratory infections, and those with neurological or psychiatric disorders impairing communication were ineligible.

### Randomization and blinding

This study adopted a randomized, double-blind design. Participants, the data recorder, and the data analyst were blinded to group assignments. Randomization was performed in a 1:1 ratio using a computer-generated sequence created by an independent researcher in Excel 2010 (Microsoft Office). Group allocation—ciprofol group (Group C) or propofol group (Group P)—was concealed in sequentially numbered envelopes opened by nurses who were not involved in patient management or data analysis.

### Study design and procedure

This was a single-center, randomized, parallel-group, active-controlled, non-inferiority trial conducted at Heyuan People's Hospital to compare the efficacy and safety of ciprofol versus propofol in patients undergoing minor gynecologic surgery. A total of 342 patients were randomized equally to the ciprofol or propofol group. All participants fasted for at least 8 h and refrained from clear liquids for at least 2 h before anesthesia induction. Each patient received an infusion of 300–500 mL of compound sodium chloride solution within 1 h before administration of the study drugs. Baseline parameters, including age, weight, height, and ASA grade, were recorded.

During surgery, continuous monitoring of HR, RR, SpO_2_, SBP, DBP, mean arterial pressure (MAP), and electrocardiogram (ECG) was performed. Oxygen was administered via mask at 6 L/min for at least 3 min before induction and maintained until full recovery.

The initial induction dose of ciprofol 0.4 mg/kg was selected based on its equivalence to propofol 2.0 mg/kg, consistent with previous studies^2–5^. Both drugs were administered intravenously within 30 s. Sedation depth was assessed using the Modified Observer's Alertness/Sedation (MOAA/S) scale, and surgery commenced when the MOAA/S score reached ≤2. If adequate sedation was not achieved within 2 min, an additional dose of ciprofol 0.2 mg/kg or propofol 1.0 mg/kg was administered intravenously over 10 s. Additional doses were allowed up to five times within 15 min; if sedation remained inadequate, crossover therapy with propofol was initiated.

### Efficacy assessments

The primary efficacy endpoint was the onset time of anesthesia, defined as the interval from the first administration of the study drug to the achievement of MOAA/S ≤2.

Secondary efficacy outcomes included: (1) anesthesia success rate, defined as the completion of surgery without the need for an alternative sedative or anesthetic (≤5 administrations within 15 min); (2) time to full alertness, defined as the time from surgery completion to the first MOAA/S score of 5 for three consecutive evaluations; (3) time to operating room discharge, defined as the time from surgery completion to a Steward score ≥4; and (4) total dosage of anesthetic agents administered.

### Safety assessments

Hemodynamic and respiratory parameters (SBP, DBP, MAP, HR, SpO_2_, and RR) were recorded at five time points: before anesthesia (T0), 2 min after induction (T1), at the start of surgery (T2), at the end of surgery (T3), and during awakening (T4). AEs were monitored throughout the study, including hypoxia (SpO_2_<95% for ≥30 s), bradycardia (HR <55 beats/min for ≥2 min), and hypotension (SBP<90 mmHg or ≥20% decrease from baseline for ≥2 min). The incidence of intraoperative body movement, injection pain, and postoperative complications—such as dizziness, postoperative nausea, and vomiting (PONV)—was documented. Satisfaction with sedation and anesthesia was assessed separately for patients and surgeons using structured questionnaires.

### Statistical analysis

Sample size estimation was based on prior clinical research involving propofol, assuming an anesthesia onset time of 1.2±0.6 min. With a significance level of 0.05, statistical power of 90%, and a non-inferiority margin of 0.2 min, 310 participants were required. Considering a 10% dropout rate, 342 patients were ultimately enrolled.

Statistical analyses were performed using Statistical Package for the Social Sciences Statistics version 25.0 (IBM, USA). Continuous variables were expressed as mean±standard deviation (SD) and compared using the unpaired t-test or Wilcoxon rank-sum test, as appropriate. Repeated-measures Analysis of Variance was applied for hemodynamic parameters. Categorical variables were presented as frequencies or percentages and analyzed using the chi-square test or Fisher's exact test. A p<0.05 was considered statistically significant.

## RESULTS

### Baseline characteristics of enrolled patients

**Figure 1:**
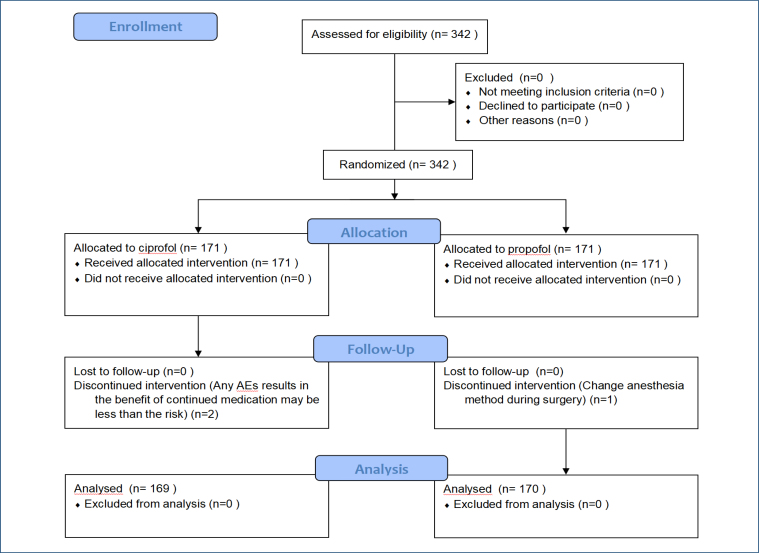
Flow diagram of the CONSORT.

A total of 342 patients were screened for eligibility. One patient in the propofol group was excluded due to a change in anesthesia method during surgery, and two patients in the ciprofol group were excluded owing to unfavorable risk–benefit assessment. Ultimately, 339 patients completed the study between August 1, 2022, and August 31, 2023 (Figure 1). Baseline demographic and clinical characteristics were comparable between the two groups. The mean age was 42.0±10.0 years in the propofol group and 43.4±9.5 years in the ciprofol group. Average height and weight were 157.1±4.6 cm and 56.9±7.6 kg in the propofol group, and 157.5±5.0 cm and 57.6±7.4 kg in the ciprofol group, respectively. Regarding ASA classification, 80.6 and 73.4% of patients were ASA I in the propofol and ciprofol groups, respectively, with the remaining 19.4 and 26.6% classified as ASA II. No statistically significant differences were observed between the groups.

### Primary outcomes

The primary endpoint was the onset time of anesthesia. The mean onset time was 37.2±10.4 s in the ciprofol group and 38.7±11.2 s in the propofol group (p=0.214), indicating no significant difference between treatments (Table 1).

**Table 1:** Summary of secondary endpoints and adverse events between the two groups.

Variables		Propofol (n=170)	Ciprofol (n=169)	p-value
The onset time of anesthesia (s)		38.7±11.2	37.2±10.4	0.214
Fully alert time (min)		3.8±1.3	4.6±1.4	<0.001
Time out of the operating room (min)		8.1±3.1	8.5±2.0	0.112
Top-up doses required (mg)		359.6±164.6	68.5±31.7	<0.001
Body movement, n (%)		26 (15.3%)	4 (2.4%)	<0.001
Patients satisfaction				
	Satisfaction	121 (71.2%)	166 (98.2%)	<0.001
	General satisfaction	49 (28.8%)	3 (1.8%)	
	Dissatisfaction	0 (0.0%)	0 (0.0%)	
Surgeons satisfaction				
	Satisfaction	151 (88.8%)	169 (100%)	<0.001
	General satisfaction	19 (11.2%)	0 (0.0%)	
	Dissatisfaction	0 (0.0%)	0 (0.0%)	
Adverse events, n (%)				
	Hypoxia	19 (11.2%)	4 (2.4%)	0.001
	Respiratory depression	6 (3.5%)	0 (0.0%)	0.040
	Bradycardia	0 (0.0%)	0 (0.0%)	1.000
	Hypotension	70 (41.2%)	17 (10.1%)	<0.001
	Pain on injection	62 (36.5%)	0 (0.0%)	<0.001
	Dizziness	4 (2.4%)	0 (0.0%)	0.133
	Nausea/vomiting	0 (0.0%)	0 (0.0%)	1.000

### Secondary outcomes

Both groups achieved a 100% anesthesia success rate. The time to full alertness was slightly longer in the ciprofol group (4.6±1.4 min) than in the propofol group (3.8±1.3 min; p<0.001), though the difference was clinically acceptable. The time to exit the operating room did not differ significantly (8.5±2.0 min vs. 8.1±3.1 min; p=0.112).

The total anesthetic dose was markedly lower in the ciprofol group (68.5±31.7 mg) than in the propofol group (359.6±164.6 mg; p<0.001). Regarding satisfaction, both surgeons (100 vs. 88.8%) and patients (98.2 vs. 71.2%) rated the ciprofol group significantly higher (p<0.001).

Intraoperative body movement occurred in only four patients (2.4%) receiving ciprofol compared with 26 patients (15.3%) in the propofol group (p<0.001), reflecting improved surgical conditions with ciprofol.

### Safety assessments

Pain on injection was reported in 36.5% of propofol patients but in none of the ciprofol group (p<0.001). Adverse respiratory and circulatory events were also less frequent with ciprofol. Hypoxia occurred in 11.2% of the propofol group versus 2.4% of the ciprofol group (p=0.001), while respiratory depression was seen in 3.5% of propofol patients and in none with ciprofol (p=0.040). Hypotension was notably lower in the ciprofol group (10.1%) compared with propofol (41.2%; p<0.001). Minor dizziness was reported by 2.4% of propofol patients, but none in the ciprofol group, and no cases of bradycardia, nausea, or vomiting occurred in either group.

### Hemodynamic changes

**Figure 2:**
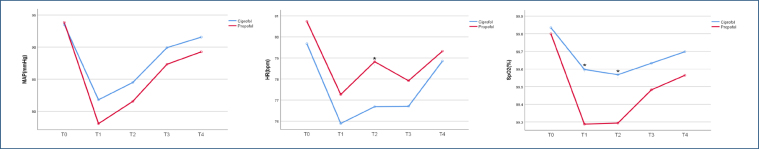
Comparisons of mean arterial pressure, heart rate, and pulse oxygen saturation (SpO_2_) between the ciprofol and propofol groups. T0: Pre-anesthesia, T1: 2 min post induction, T2: the beginning of the operation, T3: the end of the operation, T4: awakening.

Both groups exhibited transient reductions in MAP, HR, and SpO_2_ after induction (T1–T4) compared with baseline (T0) (p<0.05), though all remained within normal clinical limits. The ciprofol group demonstrated smaller fluctuations in MAP, HR, and SpO_2_ than the propofol group, indicating superior hemodynamic stability (Figure 2).

## DISCUSSION

Patients undergoing minor gynecologic surgery often experience considerable anxiety, highlighting the need for effective and safe sedative agents. This randomized trial demonstrated that ciprofol was non-inferior to propofol in anesthesia onset time during minor gynecologic surgery. Although recovery to full alertness was slightly longer with ciprofol, the delay was clinically acceptable. More importantly, ciprofol offered clear advantages, including less injection pain, fewer AEs, greater hemodynamic stability, and higher patient and surgeon satisfaction. These findings indicate that ciprofol is a promising sedative for short gynecologic procedures.

Propofol remains the most widely used intravenous anesthetic due to its rapid onset, predictable recovery, and high controllability. However, its use is frequently limited by adverse effects such as injection pain, cardiorespiratory depression, and hypotension^6^. Ciprofol, a novel phenol derivative structurally similar to propofol, has demonstrated comparable sedative efficacy but improved safety profiles. Its enhanced affinity for the GABA. A receptor, attributed to the introduction of a cyclopropyl group in its molecular structure, strengthens its anesthetic potency while reducing undesirable effects such as respiratory depression and vascular irritation.

Consistent with earlier studies, this investigation found no significant differences between ciprofol and propofol in anesthesia onset, anesthesia success rate, or operating room discharge time. The slightly prolonged recovery time with ciprofol aligns with prior findings in patients undergoing gastroscopy and colonoscopy. However, other studies have reported longer onset times for ciprofol, which may be influenced by factors such as patient age, procedure type, or concomitant sedative and analgesic use^7^.

In terms of safety, oxygen desaturation—the most frequent AE related to propofol—occurred less often with ciprofol. This observation supports previous evidence that ciprofol causes less respiratory suppression due to its distinct molecular configuration and higher receptor selectivity^8^. This advantage of ciprofol may be attributed to its pharmacological specificity: (1) Ciprofol has a 4–5 fold higher affinity for GABA receptors and can achieve the same sedative effect at lower plasma concentrations when compared to propofol; (2) Ciprofol induces respiratory rhythm disorders through direct medullary inhibition, thereby reducing inhibition of the brainstem respiratory center. Similarly, the incidence of hypotension was significantly lower in the ciprofol group, in agreement with prior multicenter studies^9^. All hypotensive episodes were transient and self-resolving, indicating favorable hemodynamic tolerance. This may be because ciprofol enhances the selectivity of GABA_A_ receptor β 3 subunit and more effectively inhibits overactivation of the sympathetic adrenal medulla system.

Higher satisfaction scores among both surgeons and patients further reinforced ciprofol's advantages. Surgeons valued the stable operating conditions and reduced body movements (2.4 vs. 15.3%), while patients appreciated the absence of injection pain (0 vs. 36.5%). These results are consistent with previous clinical reports, confirming that ciprofol's structural modification—specifically the cyclopropane substitution that forms a chiral center—enhanced hydrophobicity, reduces vascular irritation, and eliminates the characteristic injection discomfort observed with propofol. Consequently, ciprofol provides a smoother and more comfortable anesthesia experience for both patients and clinicians.

This study provided high-quality evidence by using a randomized, double-blind, active-controlled design involving a large sample of 339 patients. Standardized sedation protocols and rigorous monitoring ensured consistent data collection. The head-to-head comparison between ciprofol and propofol under identical operative conditions allowed direct evaluation of pharmacodynamic differences. Moreover, multiple clinically meaningful endpoints—including onset time, recovery, hemodynamic parameters, adverse respiratory events, and satisfaction scores—provide a comprehensive safety and efficacy profile.

Several limitations should be acknowledged. First, the study population included only ASA I–II patients aged 18–65 years; thus, findings may not be generalizable to elderly, pediatric, or high-risk patients. Second, this was a single-center trial, which may limit external validity despite the large sample size. Third, the assessment of safety of ciprofol was limited to the in-hospital phase, and future studies incorporating patient-reported outcome measures at longer-term follow-ups are warranted. Finally, although both sedation regimens followed standardized dosing, variability in individual pharmacokinetics may still influence onset and recovery.

The study's findings have direct and practical clinical relevance. Ciprofol demonstrated equivalent anesthetic effectiveness but significantly better safety, particularly regarding reduced hypotension, minimal respiratory suppression, and complete elimination of injection pain. These advantages make ciprofol especially suitable for outpatient gynecologic procedures, where rapid turnover, patient comfort, and hemodynamic stability are paramount. Improved surgeon and patient satisfaction further supports integration of ciprofol as a preferred sedative agent in ambulatory gynecologic anesthesia pathways.

## CONCLUSION

Ciprofol provides anesthetic efficacy comparable to propofol while offering markedly superior safety, stability, and patient experience during minor gynecologic surgery. Its reduced incidence of hypotension, respiratory compromise, and injection pain, combined with high satisfaction ratings, supports its use as a preferred sedative in fast-turnover outpatient gynecologic settings. Future multicenter studies across broader patient populations will help refine its clinical role.

## Data Availability

The datasets generated and/or analyzed during the current study are available from the corresponding author upon reasonable request.
